# Interplay between Host tRNAs and HIV-1: A Structural Perspective

**DOI:** 10.3390/v13091819

**Published:** 2021-09-13

**Authors:** Jinwei Zhang

**Affiliations:** Laboratory of Molecular Biology, National Institute of Diabetes and Digestive and Kidney Diseases, Bethesda, MD 20892, USA; jinwe.zhang@nih.gov

**Keywords:** HIV, tRNA, Gag, matrix, capsid, replication, reverse transcription

## Abstract

The cellular metabolism of host tRNAs and life cycle of HIV-1 cross paths at several key virus–host interfaces. Emerging data suggest a multi-faceted interplay between host tRNAs and HIV-1 that plays essential roles, both structural and regulatory, in viral genome replication, genome packaging, and virion biogenesis. HIV-1 not only hijacks host tRNAs and transforms them into obligatory reverse transcription primers but further commandeers tRNAs to regulate the localization of its major structural protein, Gag, via a specific interface. This review highlights recent advances in understanding tRNA–HIV-1 interactions, primarily from a structural perspective, which start to elucidate their underlying molecular mechanisms, intrinsic specificities, and biological significances. Such understanding may provide new avenues toward developing HIV/AIDS treatments and therapeutics including small molecules and RNA biologics that target these host–virus interfaces.

## 1. Introduction

Viruses are opportunistic parasitic pathogens that mutate and evolve at accelerated paces compared to their hosts. Their reiterative infection, replication, and transit cycles expose viral elements to the crowded, confined intracellular environment of the host, creating ample opportunities for viruses to evolve new interfaces with various host components and to usurp host resources for viral purposes. One of the most abundant (micromolar) and essential components of the host housekeeping machinery is the dynamic pool of transfer RNAs (tRNAs) that continually ferry amino acids to the ribosomes to sustain protein synthesis, cellular growth, and renewal. Host tRNAs are among the most ancient and fundamental building blocks of free-living, organic life forms. As a primary conduit between the RNA world and the protein world, tRNAs act as physical bridges and adaptors to enable RNA-directed, RNA-catalyzed protein synthesis [1,2,3,4,5,6]. Such pivotal roles of tRNAs in cellular mechanism have subjected them to constant natural selection and evolution. This is evidenced by the high percentage of tRNA nucleotides that receive post-transcriptional modifications compared to mRNAs and even rRNAs, as well as the acquisition, fixation, and propagation of complex protein enzymes dedicated to fine-tuning tRNA structure and functions [7,8,9,10,11,12,13,14,15]. Compensating for the lack of extraordinary lengths of evolution time experienced by tRNAs, HIV-1 and other retroviruses undergo dramatically accelerated evolution, owing to their rapid replication cycles and high mutation rates of their reverse transcriptases.

The canonical adaptor function of tRNAs dictates that they remain largely free and available to shuttle between aminoacyl-tRNA synthetases (aaRSes), which install the amino acids, and the ribosomes that consume the amino acid cargos and eject depleted tRNAs [12,13,14,16,17]. In order to transit the ribosome from the A to P to E sites, tRNAs cannot stay shielded by cellular proteins at all times. As a result, host tRNAs largely exist as abundant, solvent-exposed molecules that feature a well-defined “L”-shaped overall architecture, characteristic molecular and electrostatic surfaces and crevices, and are critically important to sustain the host metabolism. These properties together make tRNAs attractive and vulnerable targets to be manipulated and exploited by viruses to serve their own purposes. In reciprocity, tRNAs and their associated protein enzymes also impact HIV-1 in profound ways [18]. As a result of this constant interchange, viruses have evolved strategies to commandeer and exploit host tRNAs for both structural and regulatory functions. In this review, I discuss recent progress in understanding these viral strategies at the expanding host–virus interfaces between tRNAs and HIV-1, primarily from a structural perspective.

## 2. The Interwoven Paths of Host tRNAs and HIV-1

**Figure 1:**
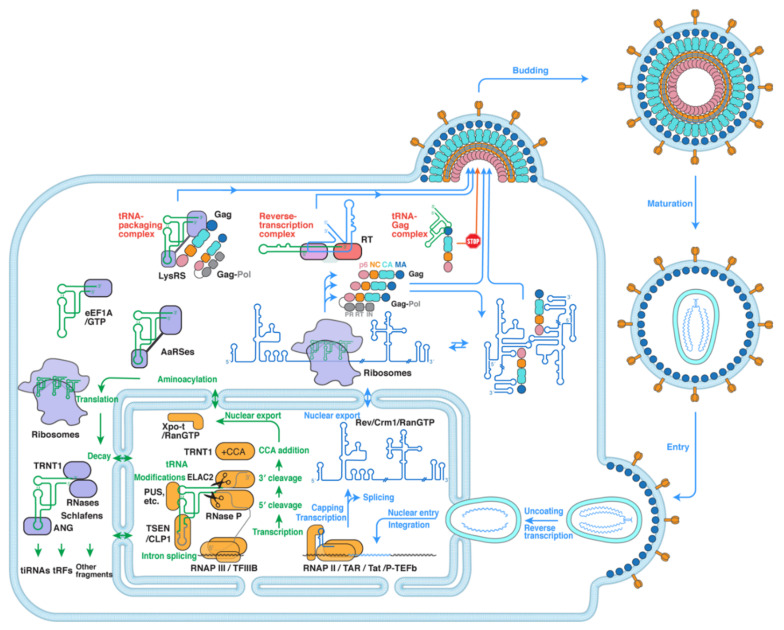
A simplified overview of the interplay between tRNA metabolism and HIV-1. tRNA metabolism (green arrows) starts with transcription by the RNAP III/TFIIIB complex [19,20] and proceeds to processing by RNase P and ELAC2 (RNase Z) [21,22,23,24,25], CCA addition by TRNT1 [26,27,28], optional intron splicing by TSEN/CLP1 [24,29], various post-transcriptional modifications such as pseudouridylation by pseudouridine synthases (PUS) [7,30], followed by nuclear export by the Exportin-t/RanGTP complex [31,32]. In the cytoplasm, tRNAs are aminoacylated by aaRSes [12,14], transported by eEF1A to the ribosomes for translation [38], and undergo cleavage and decay by various RNases to produce tRNA fragments for further gene regulation [33,34,35,36,37]. The three green tRNAs inside the ribosomes denote the E-, P-, and A-site tRNAs transiting the ribosomes. HIV-1 virions (following the blue arrows) fuse with the plasma membrane of infected cells and release their conical capsids [39], which then travel to the nucleus while undergoing reverse transcription [40,41,42], engage the nuclear pore complex, pass through, and uncoat to release the nascent double-stranded DNA (dsDNA) genome for integration [43,44]. Proviral DNA transcription initiates with RNAP II and switches to productive elongation with the stimulation by TAR RNA, HIV-1 Tat protein, and the super elongation complex [45,46]. Then, HIV-1 RNA (in blue) is spliced or exported to the cytoplasm by the Rev/Crm1/RanGTP complex [47,48,49,50,51], and it can assume a monomeric form to template translation or a dimeric form to be packaged into new virions [52,53]. Finally, viral particles assemble and bud from the infected cell and mature into infectious virions [54,55,56]. This review highlights three interfaces and complexes formed between host tRNAs and HIV-1 (highlighted in red): namely, the reverse transcription complex [57], tRNA–packaging complex [58], and tRNA–Gag complex [59].

To understand how host tRNAs and HIV-1 cross paths, we need to first understand their individual trajectories in the cell (Figure 1). Human tRNAs start their life cycle as pre-tRNA transcripts produced by RNA polymerase (RNAP) III recruited to internal promoters by transcription factors TFIIIB and TFIIIC [19,20]. Then pre-tRNAs are sequentially processed by two endonucleases: first the RNase P ribozyme to remove the 5′-leader and then ELAC2 (RNase Z) to trim its 3′-trailer, which is dependent on the La protein associated with its 3′ terminal oligo-uridine region [21,22,23,24,25]. Then, the CCA-adding enzyme TRNT1 appends a universal 3′-CCA trinucleotide, producing mature tRNAs [26,27,28]. Some intron-containing pre-tRNAs are also spliced by the TSEN/CLP1 complex [24,29]. Nearly all tRNAs receive extensive modifications such as pseudouridylation by pseudouridine synthases (PUS) [7,30], before they are licensed for nuclear export by Exportin-t/RanGTP [31,32]. Once in the cytoplasm, tRNAs are aminoacylated by their respective aaRSes and relayed to eEF1A, which brings the aminoacyl-tRNAs to the ribosomes for translation [12,14,33]. Although tRNA decay pathways are increasingly understood in lower eukaryotes such as *Saccharomyces cerevisiae*, much less is known about tRNA decay in human cells [33,34]. One tRNA-decay pathway involves the stress-activated tRNA ribonuclease angiogenin (ANG) and possibly additional nucleases. ANG cuts tRNAs in the anticodon loop and produces tRNA fragments termed tiRNAs (tRNA-derived stress-induced RNAs, or tRNA halves). Progressive cleavage, or additional nucleases create even shorter fragments called tRFs (tRNA-derived fragments; about a quarter-size of the full tRNA). tiRNAs and tRFs are suggested to play regulatory roles such as translation repression [35,36,37].

Upon membrane fusion of infectious HIV-1 virions with host cells, cone-shaped viral capsids are released into the cytoplasm and are actively transported to the nucleus via the host microtubule and actin cytoskeleton [39,40,41,42,56]. At the same time, reverse transcription of the single-stranded RNA (ssRNA) genome to double-stranded DNA (dsDNA) occurs, largely in newly infected cells [60]. Then, capsids enter the nucleus via the nuclear pore complex (NPC) and uncoat to release the reverse-transcribed dsDNA poised for genome integration [43,44]. HIV-1 proviruses can stay latent and replication-competent for 10–15 years prior to reactivation [61]. For activation, HIV-1 proviral DNA is transcribed by host RNAP II, which initially produces mostly short 5′ truncated transcripts and then pauses until viral Tat protein is produced. The accumulation of Tat allows it to bind the 5′ TAR (*trans*-activation response) RNA and to recruit host positive transcription elongation factor b (P-TEFb) as part of the super elongation complex [45,46]. The action of this ribonucleoprotein complex releases paused RNAP II near the promoter and dramatically stimulates transcription elongation to generate full-length (9.2 kilobase, or kb) genomic RNAs. To export this intron-containing, unspliced or partially spliced RNA to the cytoplasm, another oligomeric ribonucleoprotein complex assembles between HIV-1 Rev protein and a *cis*-acting RRE (Rev response element) RNA element in the viral genome [47,48,49,50,51].

Once in the cytoplasm, the genomic RNA is suggested to assume at least two structural forms which lead to two distinct fates, as determined by the transcription start site (TSS) heterogeneity on its 5′ end [52,53]. Viral RNA genomes with shorter 5′ ends (e.g., “capped 1G”, meaning harboring a single G on the 5′ end that is further capped) juxtapose and coaxially stack the termini of the 5′ TAR hairpin with its ensuing polyA hairpin, which in turn exposes the DIS (dimerization initiation sequence) palindrome to form dimers destined for packaging into new virions [53]. By contrast, viral RNA genomes with slightly longer 5′ ends (e.g., capped 2G or 3G, meaning transcripts that harbor 2–3 Gs and the 5′ cap) are unable to stack with and presumably stabilize the polyA hairpin, causing the metastable hairpin to collapse. This leads to sequestration of the DIS sequence in a stem, and thus, the HIV-1 RNA assumes a monomeric form fated to serve as efficient translation templates. Then, viral translation produces HIV-1 Gag polyprotein and about a dozen other viral proteins. Gag is the conductor that orchestrates multiple steps and threads of viral particle assembly. It is responsible for specifying assembly sites on the plasma membrane, the recruitment and packaging of dimeric genomic RNA, tRNA primers, and proteins, the incorporation of envelope glycoprotein (Env), etc. [54,55]. Indeed, Gag expression alone is sufficient to drive the assembly of virus-like particles that are morphologically indistinguishable from functional immature virions [54,62]. Upon budding, HIV protease (PR) cleaves the radially arranged Gag polyproteins in virions to release its individual domains (matrix or MA, capsid or CA, nucleocapsid or NC, and p6). These mature proteins act coordinately to assemble fullerene-like conical capsids around the NC-bound dimeric genomic RNA and ultimately confer infectivity to the now mature virions [56].

As can be seen above, host tRNAs and HIV-1 elements share the same space and time in all three locations including the cytoplasm, nucleus, and virions during essentially all stages of the HIV-1 life cycle. This provides ample opportunities for potential encounters and the evolution of new interfaces. While most of our current knowledge on tRNA–HIV-1 interactions is limited to the cytoplasm, it is conceptually conceivable that tRNAs and pre-tRNAs may engage in presently unidentified, functionally important interplay with HIV-1 components in the nucleus and in virions. In the following three sections, the molecular structures and biological functions of three significant tRNA–HIV-1 complexes will be discussed, namely the reverse transcription initiation complex (RTIC) responsible for genome replication [57], the tRNA–packaging complex that captures and encapsulates tRNA primers for reverse transcription [58,63,64], and the tRNA–Gag complex that regulates Gag membrane localization and virus assembly [59,63].

## 3. Exploitation of tRNA as Reverse Transcription Primers

The most important role that host tRNAs play for HIV-1 and most other retroviruses is to prime the reverse transcription of the RNA genome. This obviates a need for HIV-1 to evolve a separate viral RNA primer besides its ssRNA genome or to commandeer a host primase to lay down the RNA primer. Retroviruses exploit the same tRNA terminal 3′-OH group used by the host for aminoacylation and peptidyl transfer as the recipient of deoxyribose nucleotides as catalyzed by reverse transcriptase (RT). HIV-1 RT is a heterodimeric enzyme composed of a longer p66 subunit and shorter p51 subunit derived from the proteolysis of p66 by HIV-1 protease (PR). While p51 plays supporting structural roles, p66 hosts both DNA polymerase and RNase H activities on its two subdomains, which coordinately create the DNA strand and destroy the RNA strand, converting the ssRNA genome to a linear dsDNA intermediate ready for nuclear import and host genome integration by HIV integrase (IN). Interestingly, tRNA^Lys3^ is further found to drive a conformational change in the p66/p66 homodimer to facilitate PR cleavage and maturation of the functional p66/p51 heterodimer [64,65]. Consistent with this role of tRNA^Lys3^ in RT maturation, knocking down Lysyl-tRNA synthetase (LysRS) in cells led to a pronounced reduction in viral particle production [65]. Given recent findings that tRNA^Lys3^ also controls virus assembly by regulating Gag membrane localization [59,63,66], the observed replication defect was likely attributable to several tRNA functionalities.

**Figure 2:**
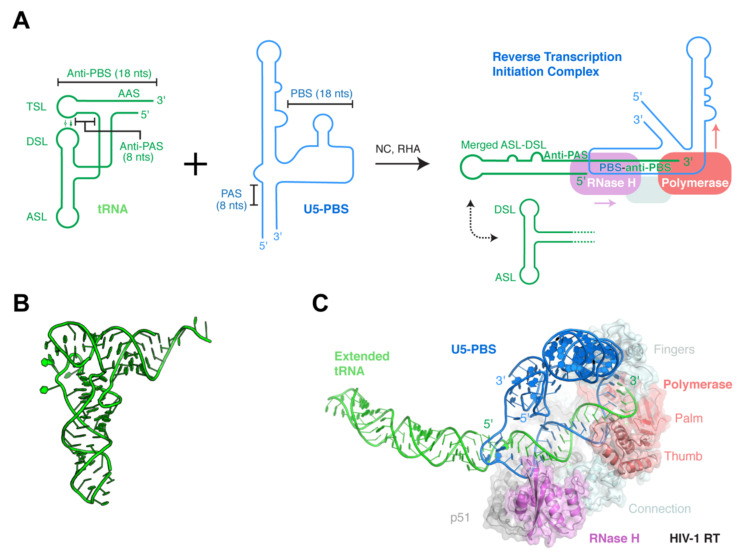
Formation and structure of the reverse transcription initiation complex (RTIC). (**A**) Secondary structure scheme of tRNA^Lys3^ (left; in green), HIV-1 U5-PBS element (middle; in blue), and resulting complex bound by RT. ASL: anticodon-stem loop; DSL: D-stem loop; TSL: T-stem loop; AAS: amino-acid accepting stem. PBS: primer-binding site; PAS: primer activation signal. In addition to the merged ASL–DSL conformation, additional conformations could be sampled, such as the one below where the ASL and DSL remain as separate hairpins. The dotted bidirectional arrow denotes this potential conformational change. Colored arrows indicate RT movements in subsequent steps of primer extension. RHA: RNA Helicase A. (**B**) Tertiary structure of tRNA^Lys3^. PDB: 1FIR. (**C**) Cryo-EM structure of the RTIC, colored as in (**A**). EMDB: EMD-7032 [57].

The assembly of functional reverse transcription initiation complexes (RTICs) is a multi-step process that only completes after virion maturation and requires the molecular chaperone activity of nucleocapsid (NC) and is assisted by RNA Helicase A (RHA) [67,68,69,70,71,72]. A recent cryo-EM structure of this dynamic ternary complex consisting of RT, the HIV-1 U5-PBS (primer-binding site) region, and tRNA^Lys3^ reveals a dramatic structural rearrangement of the tRNA^Lys3^ primer (Figure 2) [57]. The cloverleaf tRNA secondary structure completely refolds into a single extended hairpin, which is characterized by the transfer and annealing of its 5′-terminal strand with the 5′ strand of the T stem. This latter region of the tRNA is also known as the anti-PAS (primer activation signal [73]), as it is complementary to the PAS sequence in the U5-PBS leader region (Figure 2A) [74]. The reshuffle of tRNA structure displaces the 18-nt-long, 3′-terminal anti-PBS element of the tRNA, making it available to base pair with the complementary PBS element on the viral RNA (Figure 2A). Furthermore, the D-stem loop (DSL) and anticodon-stem loop (ASL) of the tRNA merge to form a distal single hairpin. This remarkable structural rearrangement is potentially stabilized by a multi-segment, elongated coaxial stack, joining the PBS–anti-PBS stem and tRNA 5′-strand–anti-PAS stem, and it is capped by the merged DSL-ASL stem loop (Figure 2B,C). This tripartite co-coaxial stack, at an impressive 45-base pair (bp) combined length, is reminiscent of the 32-bp-long coaxial “central spine” formed by the U-shaped T-box riboswitches clamping their tRNA substrates [75,76,77,78,79]. As is in the case of the T-boxes, this elongated coaxial stack may provide requisite stabilizing energy to help offset the substantial cost of disrupting both individually stable structures of the U5-PBS template and tRNA primer. Interestingly, this RTIC structure is clearly dynamic and will likely further rearrange into downstream complexes involving another tRNA refolding event, which will be needed to enable the PAS–anti-PAS pairing. Thus, the complex choreography of the tRNA primer required for forming several conformationally and topologically distinct RT complexes may have imposed the specificity for distinct tRNA primers used by different retroviruses. Notably, tRNA^Lys1^ and tRNA^Lys2^, isoacceptors of tRNA^Lys3^, are similarly packaged into HIV-1 as tRNA^Lys3^ due to comparable interactions with LysRS, but they are not used as RT primers, which is presumably due to 14–16 nts differences in the sequences, including one in the anticodon [58].

## 4. Hijacking and Packaging of tRNA Primers into HIV Virions

HIV-1, as other retroviruses, not only packages two copies of its genomic RNA and its required tRNA primers but also a number of other host noncoding RNAs [68]. Interestingly, most of these host ncRNAs are nascent RNAP III transcripts exported into the cytoplasm, which are minimally processed and unbound by their protein partners such as La. A comprehensive RNA “packageome” analysis [80], together with other targeted searches revealed that the 7SL RNA, a component of the signal recognition particle (SRP), is the most abundant host RNA packaged into HIV-1 virions. The packaging of 7SL is distinct from that of the viral genome, and it is mediated by direct interactions with Gag both in the cytosol and on the plasma membrane [81]. The packaging of 7SL RNA was suggested to facilitate the co-packaging of antiviral cytidine deaminase APOBEC3G as a co-factor [82]. In general, the role of most packaged host ncRNAs in the HIV life cycle remains unclear.

**Figure 3:**
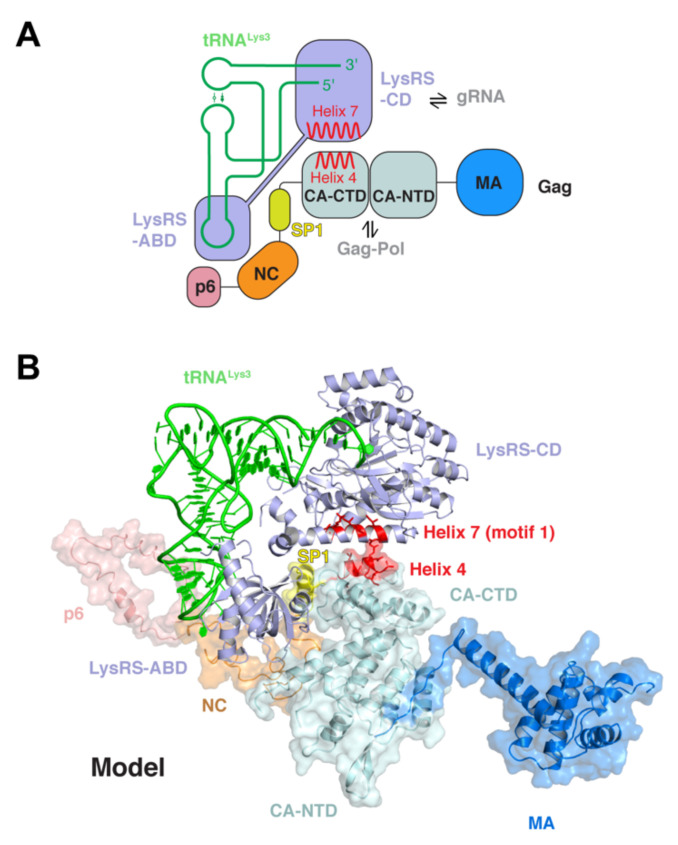
Hypothetical structural model of a tRNA-packaging complex. (**A**) Cartoon scheme of a proposed tRNA-packaging complex consisting of tRNA^Lys^, LysRS (Lysyl-tRNA synthetase), Gag, HIV genomic RNA, and Gag-Pol. ABD: anticodon-binding domain; CD: catalytic domain. NTD: N-terminal domain; CTD: C-terminal domain. (**B**) A hypothetical structural model of the tRNA-packaging complex, which was modeled based on known interactions between Gag and LysRS (in red) and between tRNA and LysRS. Gag-Pol and genomic RNAs are not shown as their locations and contacts are less clear.

By contrast, the packaging of specific tRNA primers by HIV-1 is mediated by a proposed five-membered packaging complex consisting of tRNA^Lys3^, LysRS, Gag, Gag-Pol precursor polyprotein, and viral genomic RNA (Figure 3) [58,83]. tRNA^Lys3^, the cargo, is recruited into virions via its direct interaction with its cognate synthetase LysRS, whose own encapsulation is turn driven by both Gag and Gag-Pol. A second direct interaction between the C-terminal domain of CA (helix 4) and the motif 1 of LysRS is necessary to recruit LysRS (Figure 3), while a third direct contact is suggested between tRNA^Lys3^ and the thumb structure of the RT domain of Gag-Pol [84,85,86,87]. This putative packaging complex is likely further stabilized by additional contacts between Gag and Gag-Pol as well as between LysRS and genomic RNA. The latter interaction involves a tRNA-like element (TLE) of the PBS region, which mimics the ASL of tRNA. Thus, this LysRS–TLE interaction competitively weakens or dissociates the recruited LysRS-tRNA^Lys3^ complex in order to hand over the tRNA primer from LysRS to the PBS for annealing [88,89,90]. Thus, HIV-1 goes to great lengths to recruit, encapsulate, and position tRNA^Lys3^ primer onto the genomic RNA, which is poised to initiate reverse transcription.

## 5. Viral Appropriation of Host tRNAs to Regulate Gag Localization

**Figure 4:**
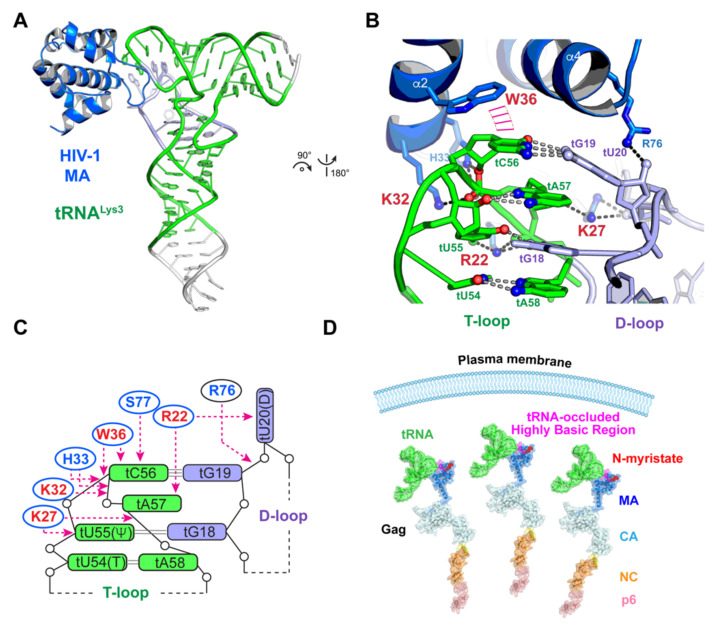
Structural basis of host tRNA regulation of HIV-1 Gag localization and replication. (**A**) Co-crystal structure of an HIV-1 MA (blue)–tRNA^Lys3^ (green) complex. PDB: 7MRL [59]. (**B**) A highly specific interface between MA and the elbow region of tRNA. Four key residues are highlighted in red. (**C**) Cartoon illustration of the MA–tRNA elbow interface. (**D**) Host tRNA binding to the N-terminal globular head of MA occludes the highly basic region (HBR) and delays Gag localization to the plasma membrane.

Interestingly, tRNAs are not only hijacked and removed to serve as essential structural components of functional virions, they are also exploited in the cytosol as regulatory molecules to control Gag localization and the timing of virion biogenesis (Figure 4) [59,65,91]. This second tRNA parasitism is mediated by a specific interaction between the N-terminal MA domain of Gag and a set of host tRNAs, as revealed by a co-crystal structure of the MA-tRNA^Lys3^ complex and in-cell crosslinking analyses [59].

MA is responsible for driving Gag localization to the plasma membrane and achieves this through the combined action of two surface features of its globular head—a 14-carbon, aliphatic myristoyl post-translational modification on its N-terminus and an adjacent highly basic region (HBR) [91,92,93,94,95,96,97,98,99]. The myristoyl group can be either exposed on the surface or be tucked inside a hydrophobic crevice on MA [100]. As the intracellular local concentrations of Gag rise, Gag multimerizes through its CA and NC domains, and the multimerization drives the exposure of the myristoyl group and facilitates its membrane insertion [100,101,102]. While this myristoyl switch is necessary to control Gag membrane binding, interactions between the HBR with plasma membrane are also essential. A molecular dynamics simulation study indicated that the HBR interactions alone are sufficient to keep MA anchored to the plasma membrane [103]. HBR–plasma membrane interactions involve both nonspecific electrostatic interactions and specific contacts between the HBR residues and phosphatidylinositol-4,5-bisphosphate (PIP_2_), which is a plasma membrane resident phospholipid [94,104,105]. Remarkably, the globular head of MA has evolved a specific structural determinant, consisting primarily of three basic (R22, K27, K32) and one aromatic (W36) residues, that acts to recognize the “elbow” structure of host tRNAs (Figure 4) [59]. The tRNA binding site overlaps significantly with the PIP_2_-binding site on MA [91], which readily explains the observed competition between tRNAs and membranes for MA binding [68,91,93,106]. This novel HIV-1–host interface produces a *K*_d_ of ≈270 nM, and considering the micromolar concentrations of tRNAs, it is expected to drive substantial tRNA occupancy on Gag. Indeed, the tRNA–MA interaction keeps substantial quantities of Gag molecules free in the cytosol and delays their association with the plasma membrane. The disruption of tRNA interactions with MA led to premature binding of Gag to the plasma membrane and significantly reduced HIV-1 replication [59]. The need for HIV-1 to delay Gag localization by exploiting host tRNAs may be to synchronize particle assembly with other necessary threads, such as more complete viral protein translation, packaging of viral genome and tRNA primers, or the removal of restriction factors such as APOBEC3G, etc. [107].

## 6. Role of tRNAs and Associated Proteins in Host Defense against HIV-1

The sections above largely described how HIV-1 seizes and manipulates host tRNAs for viral gains. In reciprocity, tRNAs and associated proteins also play prominent roles in mounting countermeasures against HIV-1, primarily at the levels of translation control. HIV-1, similar to other lentiviruses, exhibit a significant bias for the usage of A-rich codons, especially in the late genes, whose translation therefore depends on certain rare tRNAs [104,105]. This A/T-rich bias is thought to help maintain genomic RNA structural flexibility. Manipulation of the host tRNA pool is a recurring viral strategy to optimize the translation of late genes, and it is also a vulnerability that can be exploited by the host to restrict viral translation. The interferon-inducible Schlafen (SLFN) family proteins, some of which bind tRNAs, are emerging new players in tRNA-mediated antiviral defense [106,108]. SLFN11 associates with host tRNAs and counters the changes in tRNA pools elicited by HIV-1 [109]. SLFN8 and SLFN13 cleave tRNAs in the 3′ acceptor stem to reduce global tRNA pools, thereby restricting HIV-1 translation [110]. By contrast, SLFN2 binds to and protect tRNAs from angiogenin-mediated cleavage, thus reducing product tiRNA-mediated translation inhibition in T cells [36]. Similar to SLFN11, human Hili and mouse Mili proteins sequester rare tRNAs preferred by HIV-1, thus exacerbating the codon non-optimality of viral genes to suppress HIV-1 translation [111].

A large number of aaRSes, including LysRS, have evolved non-canonical functions in a variety of cellular processes beyond translation, which may include antiviral functions [16,18]. GCN2, the founding member of integrated stress response, harbors a Histidyl-tRNA synthetase (HisRS)-like domain that bind tRNAs [112,113]. Interestingly, GCN2 is suggested to be activated by HIV-1 and other viral RNAs, and it restricts both viral translation by eIF2α phosphorylation and genome integration by phosphorylating IN [114,115]. Given the recent expansion of known GCN2 targets in cells [116], additional HIV-1-related genes may cross paths with GCN2. Running in parallel to GCN2 is PKR, an interferon-induced antiviral eIF2α kinase, which restricts and is antagonized by HIV-1 through multiple mechanisms [117,118,119].

## 7. Summary, Conclusions and Outlook

Retroviruses such as HIV-1 encapsulate RNA genomes, which encode RNA-binding proteins that in turn associate with, organize, and prepare the genomes for packaging into new virions. The nuclear and cytosolic presence of both viral RNAs and virally encoded RNA-binding proteins allow them to intermingle with host RNAs and RNA-binding proteins, which prominently feature host tRNAs and tRNA-associated protein machineries that support translation or regulate cellular metabolism. Through evolution, new virus–host interfaces have gradually emerged between viral RNAs and host proteins (e.g., HIV-1 TAR and P-TEFb [45], TAR and PKR [120,121], PBS and RHA [67]), viral proteins and host RNAs (e.g., Gag matrix and tRNA [59,63]), viral RNAs and host RNAs (e.g., PBS and tRNA primer [57,69]), and viral proteins and host proteins (e.g., Gag and LysRS [63,87,88]). It is interesting that HIV-1 has developed several key dependencies on free host tRNAs, in addition to usurping the translation machineries through which tRNAs traverse. In addition, the host and HIV-1 diverge substantially in their structural, metabolic, and regulatory characteristics and needs, such as codon preferences and mRNA structures. These distinctions create opportunities for viruses to suppress the host metabolism and divert limited host resources toward producing more viruses, and for hosts to selectively restrict the viruses while sparing their own metabolism. This is exemplified by the fight over tRNA pools between HIV-1 and the host Schlafen proteins. Therefore, newly evolved virus–host interfaces, especially those between RNAs and proteins, provide a powerful new arsenal that can be leveraged by both viruses and hosts to serve their distinct objectives.

While significant progress has been made in the past few years to visualize several key HIV-1–tRNA interfaces and to understand their molecular specificities, much remains incompletely understood and warrants further investigation. For the reverse transcription complex, additional higher-resolution structures that represent downstream events are expected to bring crucial insights into the dynamic conformational changes in both the tRNA primers and the PBS. For HIV-1 MA–tRNA interactions, the tRNA selectivity by MA is dependent on the sequence, structure, and potentially also flexibilities of the tRNA D-loop [59]. A full accounting of the precise tRNA-binding preferences of MA and how MA recognizes several in vitro selected RNA aptamers still awaits further examination [68,122,123]. In addition, mature MA is presumably released into the cytoplasm upon new infections. It is unknown if these MA molecules would associate with host tRNAs and impact translation or the tRNA pool. For the large tRNA-packaging complex, no structure is yet available. Recent technical advances in cryo-electron tomography (cryo-ET) provide an exciting new avenue to visualize such large molecular assemblies in situ [124]. In addition to these known tRNA–HIV-1 interfaces, others likely exist and remain to be discovered. For instance, potential interactions in the nucleus involving pre-tRNAs and those potentially occurring on various cell membranes have not been extensively explored. Gag from several retroviruses such as HIV-1 and Rous sarcoma virus (RSV) have been suggested to enter the nucleus [125,126]. A recent report suggested that nuclear HIV-1 Gag colocalizes with and may form complexes with unspliced genomic RNA and HIV-1 Rev, with implications in genomic RNA export and packaging [126]. Given the abundance and concentration of pre-tRNAs and tRNAs in the nucleus, Gag–tRNA interactions, especially those through the tRNA elbow [59], could play additional structural or regulatory roles. In addition, since LysRS is also present in the nucleus [122,123,127], some of the contacts that make up the tRNA-packaging complex (Figure 3) may also occur in the nucleus. It is also unclear how HIV-1 exactly manipulates host tRNA pools to optimize late-gene translation and exploits the dynamic multi-synthetase complexes (MSC) for LysRS release and potentially other tRNA-related functions [128]. While an effective HIV-1 vaccine is still well beyond the horizon, a fundamental understanding of HIV-1–host interactions continue to provide key avenues toward developing new treatments and therapeutics in our tug of war with this rapidly mutating pathogen.

## Data Availability

Not applicable.
